# Evaluating the Impact of Managed Long-Term Services and Supports on HCBS Providers: Findings from a Seven Wave Survey

**DOI:** 10.1093/geroni/igaf122.3024

**Published:** 2025-12-31

**Authors:** Heather Mentch, Keri Kastner, Howard Degenholtz

**Affiliations:** University of Pittsburgh, Pittsburgh, Pennsylvania, United States; University of Pittsburgh, Pittsburgh, Pennsylvania, United States; University of Pittsburgh, Pittsburgh, Pennsylvania, United States

## Abstract

Community HealthChoices (CHC), Pennsylvania’s managed long-term services and supports (MLTSS) program, was designed to improve service coordination, expand access to home and community-based services (HCBS), and ensure provider financial sustainability. This study presents findings from a seven-wave (2018–2024) statewide survey of HCBS providers conducted by the Medicaid Research Center at the University of Pittsburgh. The survey assessed provider experiences with managed care organizations (MCOs), satisfaction with CHC implementation, financial viability, service accessibility, and provider-MCO interactions. Response rates ranged from 19% to 49%, with data analyzed to examine regional trends and provider perspectives. Findings indicate that provider sentiment evolved over time, with initial optimism declining post-implementation before returning to or exceeding pre-implementation levels. While MCO-provider relationships strengthened, there are persistent differences in the rank ordering of perceptions of MCOs. Providers increasingly recognize the importance of upgrading technology. Providers’ perceptions of quality and access have generally improved over time, with the notable exception of a slight decrease in perceptions of quality in the Southeast. Providers’ expectations regarding financial viability initially declined post-implementation, but have generally improved over time, apart from a recent slight decrease in the Southeast. This multi-year evaluation highlights the complexities of CHC implementation from the provider perspective. While progress has been made in communication, administrative efficiency, and service accessibility, opportunities for improvement related to regulatory burden and financial stability persist. Regional differences suggest that implementation experiences vary, underscoring the need for targeted policy improvements. Future efforts should focus on strengthening provider financial stability and supporting technological improvement.

